# Does altitude increase the risk of traumatic aortic injuries? A retrospective cohort study among six level I trauma centers in the United States

**DOI:** 10.1186/s13037-022-00340-x

**Published:** 2022-09-15

**Authors:** Stephanie Jarvis, Patrick Rudersdorf, James Poling, Andreas Hennig, Kristin Salottolo, Travis Bouchard, Allen Tanner, Wendy Erickson, Sidra Bhuller, Logan Ouderkirk, Jeffrey Simpson, Kaysie Banton, Elizabeth Kim, David Bar-Or

**Affiliations:** 1 Injury Outcomes Network (ION) Research, 501 East Hampden Ave, Englewood, CO 80113 USA; 2 Saint Anthony Hospital, 11600 West 2nd Place, Lakewood, CO USA; 3 Swedish Medical Center, 501 East Hampden Ave, Englewood, CO USA; 4 Penrose Hospital, 2222 N. Nevada Ave, Colorado Springs, CO USA; 5 Research Medical Center, 2316 East Meyer Blvd, Kansas City, MO USA; 6 Wesley Medical Center, 550 N Hillside St, Wichita, KS USA; 7 Medical City Plano, 3901 West 15th Street, Plano, TX USA

**Keywords:** Traumatic aortic injuries, Altitude, Thoracic trauma, Abdominal trauma, Blunt trauma

## Abstract

**Background:**

Traumatic aortic injuries (TAIs) are rare but are associated with a high mortality. Prior studies have shown skiers and pilots, whose injuries occur at high altitudes, are at an increased risk for a TAI. The purpose of this study was to examine the effect of altitude on the incidence of TAIs across all causes of injury.

**Methods:**

This retrospective cohort study at six Level I trauma centers (8/1/2016–1/1/2020) included adult blunt trauma patients with a chest or abdomen injury. High altitude injuries (> 5000 ft.) were compared to low altitude injuries (≤ 5000 ft.). The primary outcome was incidence of TAI.

**Results:**

There were 8562 patients, 37% were at high altitude and 63% at low altitude. High altitude patients were older (*p* < 0.01), more often Caucasian (*p* < 0.01) and had a higher ISS (*p* < 0.01). There was a significantly greater incidence of TAI at high altitude than low altitude (1.5% vs. 1.1%, *p* = 0.01). The median altitude was significantly higher for patients with a TAI than for patients without a TAI (5100 ft. vs. 1400 ft., p = 0.01). After adjustment, high altitude patients had 2-fold [OR: 2.4 (1.6, 3.7)] greater odds of having a TAI than low altitude patients.

**Conclusion:**

TAIs were more prevalent among high altitude injuries. Providers should be aware of the increased incidence of TAIs at high altitudes particularly when there is a delay in diagnosis and transfer to a trauma center with appropriate resources to manage these critical injuries. TAI screening at high altitude trauma centers may improve survival rates.

## Background

Traumatic aortic injuries (TAIs) are rare, occurring in 2% of blunt trauma cases, but result in death for up to 90% of cases [1]. A vast majority of cases do not make it to the hospital due to the high on-scene mortality rate [2]. In patients who survive the initial injury and are admitted to the hospital, rapid detection and treatment of TAIs are important to improve the survival rate, as most patients die within 24 hours of injury [2, 3]. Fast deceleration, torsion, shearing forces, compression, upward thrust of the mediastinum, osseous pinch, sudden blood pressure elevation and stretching of the aorta over the spine are mechanisms thought to explain TAI [2, 4].

Demonstrated predictors for TAI include hypotension < 90 mmHg, long-bone fractures, pulmonary contusions, left scapula fractures, hemothorax, pelvic fractures, aortic intimal flap, aortic thrombus formation, multiple-trauma, widened mediastinum, and mediastinal hematomas [2, 3, 5]. In non-traumatic cases, risk factors for spontaneous aortic injuries include: older age, smoking, prior aneurysms, congestive heart failure, hypertension, diabetes mellitus,  atherosclerosis, history of cardiac surgery, congenital disorders, and inflammatory diseases [6]. Some activities such as professional sports, weight lifting, military training, skiing, and flying airplanes have been associated with both spontaneous aortic injuries and TAIs; altitude may play a role in the latter two activities [7–11]. Abnormally large aortic diameters have also been reported among pilots, which could be related to altitude [7]. High altitude has known physiological effects on the cardiovascular and respiratory systems including the following: increased heart rate, systemic hypertension, tachycardia, hypoxia, rising pulmonary vascular pressure, diminished inspiratory oxygen pressure, impairment in arterial oxygen transport, and augmented oxygen uptake [13–15].

Whether the altitude where the injury occurred is associated with developing a TAI has not been previously reported, but this potential association has great importance for physicians treating patients at high altitudes because of the high mortality rate of TAIs and need for early treatment. The purpose of this study was to compare the incidence of TAIs and characteristics of TAIs among blunt trauma injuries occurring at high and low altitude.

## Methods

This was a retrospective study including adult blunt trauma patients admitted 8/1/2016 to 1/1/2020 to six level I trauma centers with an Abbreviated Injury Scale (AIS) score ≥ 2 points for the chest or abdomen regions; penetrating trauma and patients suffering from burns were excluded. This study was approved by the following four institutional review boards (IRB) representing all six participating trauma centers with a waiver of consent: Medical City Plano IRB (Study No: 1544946), CommonSpirit Health Research Institute IRB (Study No: 1544941), HCA-HealthONE IRB (Study No: 1543632), and Western IRB (Work No: 1–1,272,926). Patients were categorized into two groups based on the altitude where their injury occurred: patients whose injuries occurred > 5000 ft above sea level will be referred to as high altitude patients and patients whose injuries occurred ≤ 5000 ft above sea level will be referred to as low altitude patients. The zip code where the injury occurred was used to assign altitudes. When the zip code where the injury occurred was missing (*n* = 1719, 20%), the initial admitting facility’s altitude was used, which could have been one of the six participating trauma centers or a different facility which later transferred the patient to one of the six participating trauma centers.

Data collected from the trauma registry included: zip code where the injury occurred, age (categorized as > 50 years old vs. ≤ 50), gender, race (Caucasian, black, Native American, Asian, or other), injury cause (categorized as fall, motorcycle / motor vehicle collision, sports accident, or other), admission vitals [presented as the proportion normal, oxygen saturation (O2, normal 96–98%), heart rate (HR, normal 60–120 beats per minute), respiratory rate (RR, normal 12–20 breaths per minute), systolic blood pressure (SBP, normal 90–150 mmHg), and Glasgow Coma Scale (GCS, normal = 15)], chest AIS (continuous), abdomen AIS (continuous), Injury Severity Score (ISS, analyzed continuously and dichotomized as major, ≥ 16, vs. minor trauma, < 16), and comorbidities [presented as proportion with the comorbidity: hypertension, bleeding disorders, smoker, diabetes, alcohol abuse, or drug use disorder; additional comorbidities were collected from the trauma registry but only the comorbidities occurring in ≥ 10% of the population were presented]. Data collected from the patient’s medical record among patients with a TAI included: the aortic diameter (analyzed continuously and dichotomized as normal vs. abnormal), location of diameter measurement, area of the aorta involved [thoracic (aortic root, ascending, proximal, mid-ascending, distal, aortic arch, isthmus, descending), abdominal (suprarenal, infrarenal)], and TAI grade (patients could have multiple grades). TAIs are classified as grade 1: intimal tear (dissection), grade 2: intramural hematoma, grade 3: aneurysm or pseudoaneurysm, and grade 4: rupture (transection) [12]. Normal diameters were defined as ≤ 21 mm (mm) for the ascending aorta, ≤ 16 mm for the descending aorta and aortic root, and ≤ 30 mm for the abdominal aorta.

The primary aim of this study was to compare the incidence of TAI among blunt trauma patients with injuries occurring at high altitude to those occurring at low altitude. The secondary aims of the study were to compare characteristics of TAI (the anatomic location of the TAI, TAI injury grade, and the aortic diameter), the mortality rate, and the cause of injury among patients with a TAI that occurred at a high altitude to patients with TAI that occurred at a low altitude.

Students t-test, Wilcoxon Rank Sum Test, Fisher’s exact test, and chi-squared tests were used for univariate analyses when appropriate. Firth logistic regression was used to determine how altitude affected the risk for TAI, as TAIs were rare. Variables which were significantly associated with the predictor (altitude) and the outcome (TAI) were included in the logistic model as confounding variables. Hosmer-Lemeshow rule on adjustment was used to determine how many variables could be included in the adjusted model. The Cochran-Armitage trend test was used to determine if altitude (categorized in 1000 ft. increments) was associated with developing a TAI. A significance level of α < 0.05 and SAS 9.4 (Cary, NC) were used to conduct all statistical analyses.

## Results

There were 8562 patients included in the study, 37% (3187) were high altitude patients and 63% (5375) were low altitude patients. As expected, and based on the definition of the groups, the median injury altitude was significantly higher among high altitude patients, 5850 ft. vs. 1000 ft., *p* < 0.0001. The two populations also differed in their demographic and admission characteristics. High altitude patients were significantly older, more often Caucasian, had higher AIS and ISS than low altitude patients. High altitude patients were more likely to have an injury caused by a fall or sports accident and were less likely to have an injury caused by a motorcycle or motor vehicle collision than low altitude patients. When further examining the sports injuries high altitude patients were more likely to have a sports injury related to a bicycle accident (42% vs 27%), or a winter sports activity (snowmobiling, skiing, snowboarding, sledding, 37% vs. 1%), and were less likely to have a sports injury related to an all-terrain vehicle (ATV, 11% vs 49%), or during horseback riding (10% vs 27%) when compared to low altitude patients, *p* < 0.0001. High altitude patients were more likely to have hypertension, a bleeding disorder, or alcoholism and were less likely to be smokers than low altitude patients. A lower proportion of high altitude patients died in-hospital than low altitude patients, *p* < 0.0001.

There were significantly more TAIs among high altitude patients than low altitude patients, 1.5% vs. 1.1%, *p* = 0.01, Table 1. The altitude where the injury occurred was significantly higher among patients who suffered a TAI than among patients who did not have a TAI, 5100 ft. vs 1400 ft., *p* = 0.01. The mortality rate was also significantly higher among patients with a TAI than patients without, 31% vs. 9%, *p* < 0.0001.

**Table 1:** Characteristics and outcomes by elevation

	High Elevation ***n*** = 3187	Low Elevation ***n*** = 5375	***p***-value
TAI, % Yes (n)	1.5% (47)	1.1% (47)	**0.01**
Age, % ≤ 50 y/o (n)	37% (1194)	52% (2814)	**< 0.0001**
Sex, % Male (n)	62% (1985)	64% (3447)	0.09
Race, % (n)			
Caucasian	85% (2607)	77% (4107)	**< 0.0001**
Black	1% (43)	12% (44)	
Native American	< 1% (12)	2% (101)	
Asian	2% (46)	1% (44)	
Other	12% (353)	9% (471)	
Cause, % (n)			
Fall	44% (1407)	25% (1361)	**< 0.0001**
MCC/MVC	33% (1060)	57% (3047)	
Sports Accident	15% (482)	3% (169)	
Other	7% (235)	15% (795)	
ISS, Median (IQR)	13 (9, 18)	12 (5, 19)	**< 0.0001**
Major ≥16, % (n)	66% (2090)	64% (3434)	0.12
Minor < 16, % (n)	34% (1097)	36% (1941)	
Chest AIS, Mean (SD)	2.6 (0.8)	2.4 (0.9)	**< 0.0001**
Abdomen AIS, Mean (SD)	2.4 (0.7)	2.0 (1.0)	**< 0.0001**
Admission Vitals, % Normal (n)^a^			
O2 Saturation	82% (2604)	67% (3595)	**< 0.0001**
GCS	82% (2603)	75% (4017)	**< 0.0001**
HR	88% (2792)	87% (4685)	**0.57**
SBP	73% (2340)	77% (4152)	**< 0.0001**
RR	79% (2514)	71% (3835)	**< 0.0001**
Comorbidities, % Yes (n)	65% (2084)	65% (3499)	0.78
Hypertension	34% (1071)	27% (1435)	**< 0.0001**
Bleeding Disorder	12% (377)	5% (264)	**< 0.0001**
Smoker	17% (540)	27% (1457)	**< 0.0001**
Diabetes	12% (369)	12% (623)	0.99
Alcoholism	8% (267)	5% (284)	**< 0.0001**
Drug Use	7% (216)	6% (308)	0.051
Mortality	4% (107)	15% (348)	**< 0.0001**

*TAI* Traumatic aortic injury, *y/o* years old, *MCC/MVC* Motorcycle collision, motor vehicle collision, *ISS* Injury severity score, *AIS* Abbreviated injury scale, *O2 Saturation* Oxygen saturation, *GCS* Glasgow Coma Scale, *HR* Heart rate, *SBP* Systolic blood pressure, *RR* Respiratory rate.^a^ = Proportions presented represent the proportion normal of patients who had the admission vital recorded, some patients’ admission vitals were missing

Variables which were independent significant predictors of TAI included: ISS, GCS (abnormal vs. normal), SBP (abnormal vs. normal), race (Caucasian vs. not Caucasian), cause of injury (Fall, MCC/WVC, sport accident, or other), alcoholism, drug use, chest AIS, and abdomen AIS. Chest AIS, abdomen AIS, and ISS were colinear and are also impacted by having a TAI, which results in higher, or worse, scores. Because of this, those variables were not included in the adjusted model for developing a TAI. After adjustment for GCS, SBP, cause of injury, race, alcoholism, and drug use, high altitude patients were 2.4 (1.6, 3.7) times as likely as low altitude patients to suffer a TAI, Table 2.

**Table 2:** Logistic regression modeling for developing traumatic aortic injuries

	OR (95% CI)	***p***-value
Low elevation	1.0 (Ref.)	Ref.
High elevation^a^	2.4 (1.6, 3.7)	**< 0.0001**
GCS (Abnormal vs. Normal)	4.7 (3.0, 7.1)	**< 0.0001**
SBP (Abnormal vs. Normal)	0.3 (0.1, 0.6)	**0.003**
Cause of Injury (Fall vs. Other)	0.3 (0.1, 0.6)	**0.0002**
Cause of Injury (MCC/MVC vs. Other)	1.4 (0.8, 2.4)	**0.02**
Cause of Injury (Sports vs. Other)	1.7 (0.5, 5.4)	0.16
Caucasian (No vs. Yes)	1.5 (1.0, 2.4)	**0.047**
Alcoholism (Yes vs. No)	1.4 (0.7, 2.7)	0.30
Drug Use (Yes vs. No)	1.7 (0.9, 3.2)	0.09

*TAI* Traumatic aortic injury, *OR* Odds ratio, *CI* Confidence interval, *GCS* Glasgow Coma Scale, *SBP* Systolic Blood Pressure, *MCC/MVC* Motorcycle collision / motor vehicle collision. ^a^ = Per Hosmer-Lemeshow Rule on confounding, up to eight variables other than elevation could be included in the model. Model fit: AUROC = 0.80. Bold values denote *p* < 0.05

**Fig. 1:**
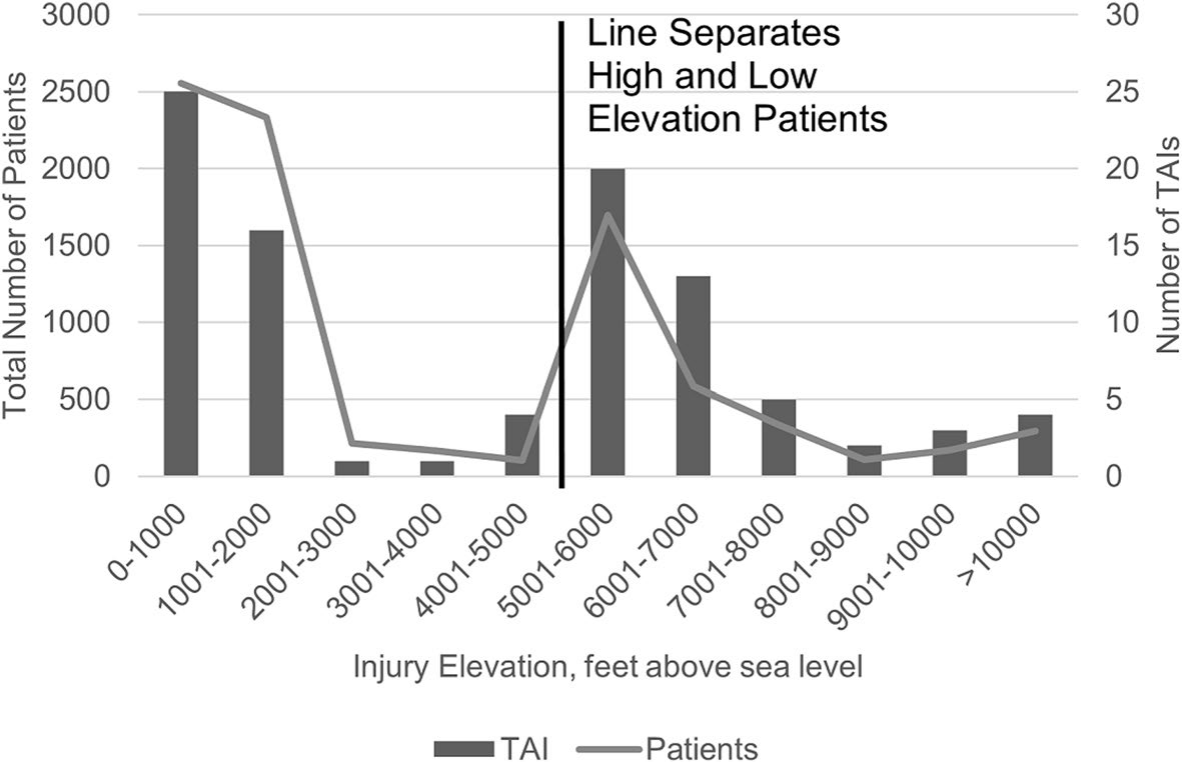
Displays the total number of patients whose injury occurred at each injury elevation, as well as the number of TAIs occurring at each injury elevation. Injury elevation was categorized into 1000-ft increments

Both the total number of patients and the number of TAIs followed a bimodal distribution with injury altitude, Fig. 1. A majority of patient’s injury occurred at 0–1000 ft. or 5001–6000 ft. above sea level. Similarly, when examined as a count, a majority of the TAIs occurred at 0–1000, 1001–2000 and 5001–6000 ft. above sea level.

**Fig. 2:**
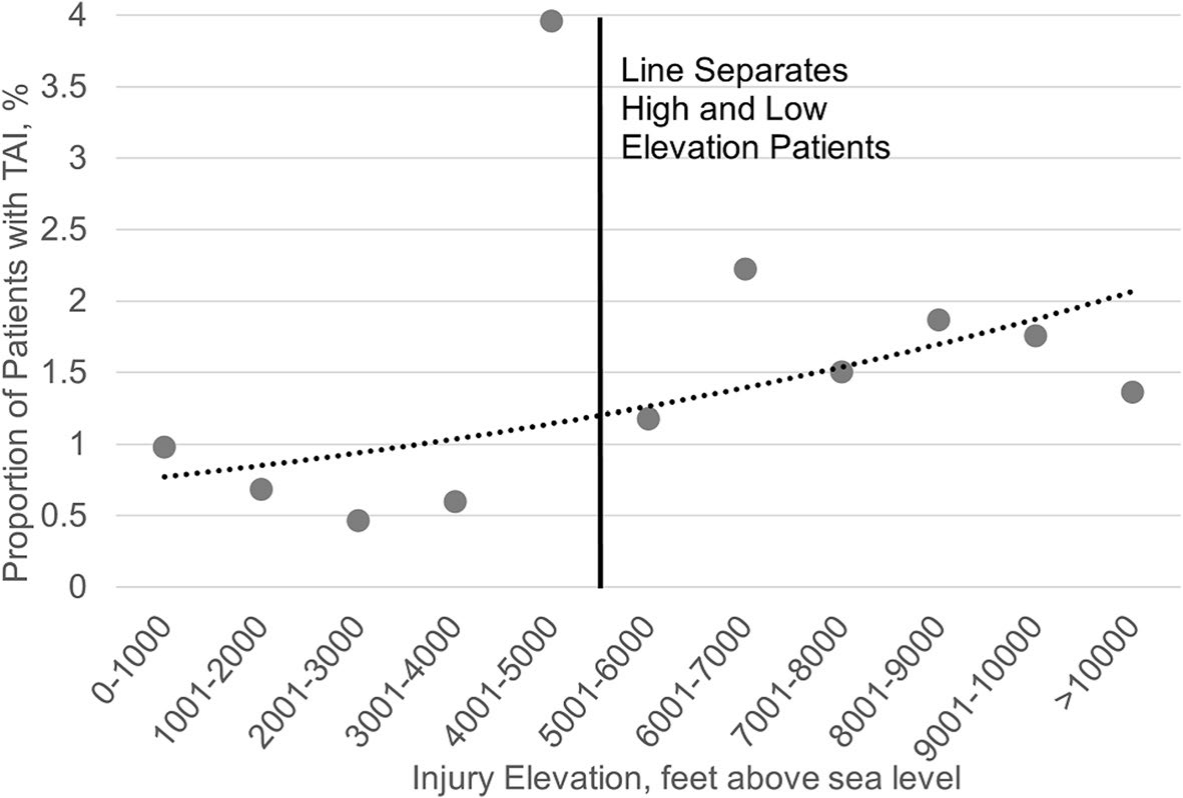
Shows the proportion of patients with a TAI among all patients whose injury occurred at each elevation category specified. Injury elevation was categorized into 1000-ft increments

When examined as the proportion of TAIs by injury altitude, a different pattern appears. As the injury altitude increased, the proportion of patients with a TAI increased exponentially, y = 0.7e^0.1x^, r-squared = 0.27, Fig. 2. The association between injury altitude by 1000 ft increments and TAI was significant, p-trend = 0.01.

Among only patients with a TAI, there were no differences in the cause of injury by altitude, *p* = 0.17, Table 3. Of the four patients who had a sports injury at high altitude and a TAI, they suffered injuries resulting from snowmobiling (*n* = 1), an ATV accident (*n* = 1) and skiing (*n* = 2). Although there were more abdominal TAIs among high altitude patients than low altitude, the proportion of abdominal aortic injuries (*p* = 0.08) and thoracic aortic injuries (*p* = 0.79) were similar between groups. The proportion of patients with a TAI in other sub-categories of each aortic region were also similar between groups. There were differences in the TAI grade between high and low altitude TAIs, *p* = 0.02. In a post-hoc analysis, the difference in grade 3 TAIs was driving the significance; thus there were significantly less Type 3 (pseudoaneurysm and aneurysm) TAIs at high altitude than there were at low altitude. Overall, the aortic diameter was greater among high altitude TAIs than among low altitude TAIs but was not statistically different. When broken down by the region where the diameter was measured, there was still no difference in the median diameter between groups. There was also no difference in the mortality rate between high altitude TAIs and low altitude TAIs, *p* = 0.93. The rate of individual comorbidities were similar between high and low altitude TAIs.

**Table 3:** TAI characteristics

	High Elevation TAIs ***n*** = 47	Low Elevation TAIs ***n*** = 47	***p***-value
Cause, % (n)			
Fall	9% (4)	6% (3)	0.17
MCC/MVC	70% (32)	72% (34)	
Sports	9% (4)	0% (0)	
Other	13% (6)	21% (10)	
Area involved, % (n)			
Thoracic Aorta	81% (28)	83% (39)	0.79
Aortic Root	2% (1)	2% (1)	> 0.99
Ascending	11% (5)	4% (2)	0.43
Proximal	4% (2)	2% (1)	> 0.99
Mid-Ascending	4% (2)	2% (1)	> 0.99
Distal	15% (7)	6% (3)	0.18
Aortic Arch	28% (13)	13% (6)	0.07
Isthmus	2% (1)	6% (3)	0.62
Descending	45% (21)	60% (28)	0.15
Abdominal	21% (10)	9% (4)	0.08
Suprarenal	2% (1)	0% (0)	> 0.99
Infrarenal	6% (3)	2% (1)	0.62
TAI Grade, % (n)^a^			
Grade 1	38% (18)	26% (12)	**0.02**
Grade 2	9% (4)	13% (6)	
Grade 3	4% (2)	28% (13)	
Grade 4	28% (13)	15% (7)	
Missing grade	11% (5)	4% (2)	
Multiple grades	11% (5)	15% (7)	
Diameter, Median (IQR), mm	30 (24, 33)	28 (23, 32)	0.72
Aortic Root	32 (31, 34)	27	N/A
Ascending	29 (25, 34)	30 (28, 32)	0.72
Descending	22 (22, 24)	25 (22, 33)	0.36
Abdominal	25 (24, 25)	^c^	N/A
Abnormal Diameter, % Yes (n)^b^	90% (27)	77% (20)	0.43
Mortality, % (n)	30% (14)	32% (6)	0.93

*MCC/MVC* Motorcycle collision / motor vehicle collision, *TAI* Traumatic aortic injury, *mm* millimeters. ^a^ = Cell chi-squared test used to determine which independent cells were driving the statistical significance; ^b^ = Proportions represent the percentage of those with a diameter recorded, some patient’s diameter measurement was not recorded in the. ^c^ None of the LE patients had a diameter recorded for the abdominal aorta. *N/A* Not applicable, statistical test not performed when there was not more than two patients in both groups

## Discussion

TAIs are extremely rare but carry a high fatality rate, resulting in considerable importance in identifying predictors of TAIs [3]. Many health conditions are associated with altitude and with TAIs, but this is the first study to our knowledge to examine the association of altitude and TAIs. TAI development was exponentially associated with increasing injury altitude; even after adjustment, high altitude patients were more than twice as likely as low altitude patients to suffer from a TAI. The mortality rate for TAI patients was three times higher than that of patients without a TAI, and that is only considering patients who survived to hospital arrival. Reducing the time to diagnosis and treatment can improve the chances of survival [2]. Screening patients with injury characteristics for TAI at high altitude trauma centers may improve survival rates. While high altitude patients had a higher incidence of TAI, high altitude did not significantly impact the aortic diameter or the risk for mortality among only patients with a TAI.

There are known physiological effects on the cardiovascular and respiratory systems at high altitudes as previously mentioned (increased heart rate, systemic hypertension, tachycardia, hypoxia, rising pulmonary vascular pressure, diminished inspiratory oxygen pressure, impairment in arterial oxygen transport, and augmented oxygen uptake) [13–15]. It is possible that these effects play a role in developing a TAI. Penning De Vries et al. hypothesized that atmospheric pressure is associated with the rupture of abdominal aortic aneurysms but failed to find a significant difference in atmospheric pressure between groups [16]. However, they did not include patients from high altitudes and the atmospheric pressure in their study was relatively constant. The authors note that because atmospheric pressure decreases with increasing altitude, they may have detected a difference in the risk for rupture if patients from other altitudes were included [16]. Reduced atmospheric pressure at high altitude could be part of the reason why grade 3 TAIs (aneurysms and pseudoaneurysms) were less common among high altitude patients and grade 4 TAIs (aortic ruptures) were more common among high altitude patients in this study.

There were factors associated with development of a TAI and high altitude including having an abnormal GCS, an abnormal SBP, the cause of injury, race, and having the comorbidities of alcoholism or drug use. A sudden dramatic increase in blood pressure specifically to the aorta and pulmonary trunk has been previously identified as a plausible explanation to the sequence of events leading to a TAI [19]. Cocaine use has also been identified as associated with development of TAI, potentially because it increases the blood pressure and heart rate, and induces vasoconstriction through synaptic stimulation [20]. This data showed that both high altitude patients, and those with a TAI, had a higher rate of alcoholism than their counterparts which is similar to a study by Ling-Yuan et al. which found that there was an increased risk for aortic aneurysms for patients who had underlying alcohol-related diseases [21]. Interestingly while those at a high altitude were more likely to have a normal GCS, having an abnormal GCS significantly increased the odds of having a TAI. Harris et al. found that non-Caucasian race was a risk factor for aortic dissection, similarly in this study, non-Caucasian race increased the risk for TAI [22]. Other studies have also shown associations between various sports activities and development of TAI [7–11]. In this study the cause of injury was a significant confounder for the association between TAI and altitude, among the patients with a TAI only those at high altitudes had sports injuries as the cause of injury. 

The mechanism behind TAI is thought to be a combination of rapid deceleration, traction, torsion, hydrostatic forces, and an osseus pinch, so it is not surprising that the cause of injury was associated with TAI development [1, 2, 4, 12, 17]. Schachner et al. identified skiers at an increased risk for aortic injuries, which may be related to the fact that skiers are often performing at high altitudes [11]. Other studies have identified activities thought to increase the risk for TAI including professional sports, weight lifting, and military training [7–9]. One case study reported a TAI after a paragliding accident [10]. However even when controlling for all of these characteristics patients at high altitudes were more than twice as likely to develop a TAI than those at low altitudes.

These finding are important because of the high mortality rate of TAIs which can be reduced through earlier diagnoses times [2]. The main cause of early death among patients with a TAI was found to be due to insufficient tissue perfusion and hemodynamic instability [3]. Missed or misdiagnosed injuries pose serious consequences; one study reported that among those who survive the initial aortic dissection, there is a lethality rate of 1–2% of patients per hour after injury [6]. TAIs often follow high-mechanism injuries which can make early identification more difficult as physicians tend to multiple injuries [3]. While early treatment has long been suggested, the European Society of Cardiology suggests a treatment approach based on the type of aortic injury [2, 3]. They suggest delayed management for patients requiring management of additional extensive injuries followed by aortic repair, but within 24 hours of the injury; whereas patients free of aortic rupture or large periaortic hematoma should be treated as emergent cases, and all others can have intervention delayed for up to 24 hours to allow for stabilization [2]. Another group suggests delayed surgery for stable patients and immediate surgery for unstable patients [18]. The need to guide treatment based on the TAI characteristics highlights the importance of identifying predictors of TAIs, such as having an injury at a high altitude. In pilots, exposure to high altitude, hypoxia and high acceleration forces can lead to exaggerated cardiovascular responses such as spontaneous aortic injuries [7]. Akin et al. examined the role of altitude on the aortic diameter in jet pilots when compared to non-jet pilots, although there was no difference between groups, the diameters reported among both groups were at least 11 mm above normal, suggesting that high altitude recreation may increase the diameter of the aorta [7]. It is possible that atmospheric pressure may also affect the aortic diameter, but in our population, there was no difference in the proportion of patients with an abnormal diameter by injury altitude. There was also no difference in the diameter of the aorta overall or by location of the diameter measurement (thoracic, abdominal, etc.). There were few patients with a recorded diameter, making comparisons of the actual measurement more difficult. The aorta diameter has shown to be related to overall patient outcomes [23–25]. Takahashi et al. found that patients admitted with an aortic dissection who needed aortic surgery, had an aortic rupture, or a dissection-related death, had a significantly larger maximum aortic diameter than patients who did not develop dissection-related events, 42 vs 34 mm [23]. Erbel et al. reported the incidence of aortic complications, such as rupture or dissection, by aortic diameter and showed that the incidence of aortic complications increased when ascending or descending aortic diameters were greater than 50 mm [24]. Another study found that a maximum diameter of the dissected aorta of ≥40 mm was associated with death by dissection or re-dissection [25]. Our study demonstrated that altitude did not significantly impact the diameter nor did it impact the mortality rate among patients with a TAI.

### Limitations

This was a retrospective study. Although the sample size was large, TAIs were rare representing under 2% of the blunt trauma patients included in this study. A large proportion of patients who suffer TAI die on scene and therefore would not be included in the trauma registries for the participating centers in this study, which were used for identification of patients. This further limited our ability to investigate the effect of altitude on all TAI, rather only those patients with a potentially treatable TAI who survive to hospital arrival. High altitude patients whose injury occurred in Colorado would likely first be admitted to a lower-level trauma center (II-V) and then transferred to one of our facilities after being stabilized; it is possible some of these patients may have died at a different facility before transfer. A study including patients from the referring centers may result in an even greater difference of TAIs by altitude. The altitude was assigned based on zip code where the injury occurred and therefore may not have been exact. The zip code where the injury occurred was missing for 20% of the patients, for those patients the altitude for the initial center was used. Other variables not investigated could have played a role in the development of TAI. The date and time of the diagnoses were not collected; time to TAI diagnosis could play a role in patient outcomes. The centers involved were not following uniform screening criteria for TAI, nor were they following uniform treatment plans.

## Conclusion

Patients whose injury occurred at high altitude were more than twice as likely to develop a TAI than patients at low altitudes. When altitude was examined continuously there was an exponential relationship with the risk for TAI. Because of the high mortality rate among TAIs, high altitude trauma centers should screen for TAIs when evaluating high-energy mechanisms or trauma patients with injuries associated with TAIs to provide more rapid diagnoses and proper treatment, which may ultimately prevent deaths.

## Data Availability

The datasets generated and analyzed during the current study are not publicly available due to our data use agreements which outline we are unable to share data with external sources. The coding methodology can be provided upon request.

## Abbreviations

TAI: Traumatic aortic injury
AIS: Abbreviated Injury Scale
O2: Oxygen saturation
HR: Heart rate
RR: Respiratory rate
SBP: Systolic blood pressure
GCS: Glasgow Coma Scale
ISS: Injury Severity Score
Ft: Feet
